# Isolation, Screening, and Active Metabolites Identification of Anti-*Vibrio* Fungal Strains Derived From the Beibu Gulf Coral

**DOI:** 10.3389/fmicb.2022.930981

**Published:** 2022-06-02

**Authors:** Bingyao Huang, Shuai Peng, Shifang Liu, Yanting Zhang, Yuxiao Wei, Xinya Xu, Chenghai Gao, Yonghong Liu, Xiaowei Luo

**Affiliations:** Institute of Marine Drugs, Guangxi University of Chinese Medicine, Nanning, China

**Keywords:** the Beibu Gulf, coral-derived fungi, anti-*Vibrio*, phylogenetic tree, equisetin

## Abstract

The Beibu Gulf harbors abundant underexplored marine microbial resources, which are rich in diversified secondary metabolites. The genera *Vibrio* is a well-known pathogenic bacterium of aquatic animals. In this study, 22 fungal strains were isolated and identified from the Beibu Gulf coral *via* the serial dilution method and internal transcribed spacer (ITS) sequence analysis, which were further divided into three branches by phylogenetic tree analysis. The crude extracts of them *via* small-scale fermentation were selected for the screening of inhibitory activity against *Vibrio alginalyticus*, *Vibrio coralliilyticus*, *Vibrio harveyi*, *Vibrio parahaemolyticus*, *Vibrio owensii*, and *Vibrio shilonii*. The results showed that eight fungal extracts displayed anti-*Vibrio* activity *via* the filter paper disk assay. Several of them showed strong inhibitory effects. Then, two tetramic acid alkaloids, equisetin (**1**) and 5′-epiequisetin (**2**), were identified from *Fusarium equiseti* BBG10 by bioassay-guided isolation, both of which inhibited the growth of *Vibrio* spp. with the MIC values of 86–132 μg/ml. The scanning electron microscope results showed that cell membranes of *Vibrio* became corrugated, distorted or ruptured after treatment with **1** and **2**. Taken together, this study provided eight fungal isolates with anti-*Vibrio* potentials, and two alkaloid-type antibiotics were found with anti-*Vibrio* effects from the bioactive strain *F. equiseti* BBG10. Our findings highlight the importance of exploring promising microbes from the Beibu Gulf for the identification of anti-*Vibrio* for future antibiotic development.

## Introduction

As an important biocenosis in marine ecosystems, a coral reef ecosystem represents an extraordinarily diverse biota in tropical environments, among which corals often constitute a dominant part of the reef biomass. Scleractinian corals harbor diverse and abundant microbial symbionts with different types of interactions, which function as the primary reef ecosystem engineers, constructing the framework and shaping the resource availability for other coral reef-associated organisms (Tang et al., 2020). Manipulation of the coral-associated microbiome was postulated as a key strategy to improve the resilience of reef-building corals. The genera *Vibrio*, known as *Vibrio coralliilyticus* and *Vibrio mediterranei*, are important coral pathogens capable of inducing serious coral damage, which has seriously impacted reef-building corals throughout the oceans as well as global warming (Esther et al., 2020). Recently, coral and its associated microorganisms have been evidenced as promising producers of structurally diverse compounds with a wide range of potent bioactivities, such as anti-inflammatory, cytotoxic, antimicrobial, antivirus, and antifouling activities (Hou et al., 2015; Sang et al., 2019).

The Beibu Gulf, located in the north of the South China Sea, harbors abundant biodiversity in both marine organisms and microorganisms and is regarded as a potential source of new species, new genes, new drugs, and new biological materials (Xu et al., 2020). However, there are relatively few reports about marine natural products from the Beibu Gulf (Carroll et al., 2019, 2020, 2021, 2022; Wang et al., 2019). In continuation of our research program aiming at the discovery of bioactive metabolites from the Beibu Gulf-derived marine fungi, a series of new bioactive compounds with diversified structures have been obtained recently (Luo et al., 2021; Lu et al., 2022; Zhang et al., 2022). Therefore, the main objective of this study was to investigate the anti-*Vibrio* potential of fungi isolated from scleractinian corals collected from the Weizhou Islands coral reef in the Beibu Gulf and to obtain and evaluate the potential exploitable anti-*Vibrio* alkaloids, equisetin and 5′-epiequisetin, from *Fusarium equiseti* BBG10.

## Materials and Methods

### Sampling and Isolation of Fungi

The corals *Porites lutea* were collected from the Weizhou Islands coral reef in Guangxi Zhuang Autonomous Region, China, in July 2019. The samples were stored in sterilized polythene bags, transported to the laboratory, and processed immediately for the isolation and cultivation of fungi. The fungi were isolated by the serial dilution method (1:10, 1:100, and 1:1,000) using potato dextrose agar (PDA) medium supplemented with sea salt (20 g/L) and chloramphenicol (20 mg/L). The inoculated plates were cultured at 25°C for 1–3 weeks and observed the growth of fungi intermittently. Fungal isolates were chosen and transferred into another blank agar plates based on their morphological traits. The isolated strains were deposited at the Institute of Marine Drugs, Guangxi University of Chinese Medicine, Nanning, China.

### Identification of Fungi

The fungal strains were cultured in PDA medium at 28°C for 5 days. The genomic DNA of the fungal strains was isolated by using the protocol described previously (Shen et al., 2020). The internal transcribed spacer (ITS) sequences were checked and amplified using ITS1-(5′-TCCGTAGGTGAACCTGCGG-3′) and ITS4-(5′-TCCTCCGCTTATTGATATGC-3′) primers. The fungi were identified mainly by analysis of the ITS sequences (as shown in Supplementary Material) in the NCBI BLAST program. The phylogenetic tree was created based on the ITS sequences by MEGA7.

### Screening of Fungi Fermentation and Extracts

The small-scale fermentations of 22 fungal isolates (BBG1–BBG22) were carried out in rice solid medium (50 g rice, 1.2 g artificial sea salt, and 60 mL H_2_O) employing with 1-L Erlenmeyer flasks at room temperature for 30 days. The fermented cultures were overlaid and extracted three times with EtOAc. All the fungal extracts (10 mg/mL dissolved in methanol) were analyzed by high-performance liquid chromatography (HPLC; Shimadzu Prominence-i LC-2030) using a PDA detector and an ODS column (YMC-pack ODS-A, 4.6 mm × 250 mm, 5 μm). In addition, the organic extracts were combined and evaporated *in vacuo* as a total crude extract for further anti-*Vibrio* assays.

### Anti-*Vibrio* Assay

All the fungal extracts, along with two isolated tetramic acid alkaloids, equisetin (**1**) and 5′-epiequisetin (**2**), were screened for antibacterial activity against *Vibrio alginalyticus*, *Vibrio coralliilyticus*, *Vibrio harveyi*, *Vibrio parahaemolyticus*, *Vibrio owensii*, and *Vibrio shilonii*, by using a K–B disk agar diffusion method (Zhang et al., 2022). The strain *Vibrio parahaemolyticus* was kindly provided by Prof. Nan Li (Nanning Normal University, Nanning, China), while other Vibrio strains were kindly provided by Prof. Chang Chen (South China Sea Institute of Oceanology, Chinese Academy of Sciences, Guangzhou, China). Each fungal extract was dissolved in dimethyl sulfoxide (DMSO) at a final concentration of 25 mg/ml, and the two compounds (**1**–**2**) were prepared at a concentration of 10 mg/ml. The positive control chloramphenicol was prepared at a concentration of 150 μg/mL in DMSO. The *Vibrio* strains were cultivated in Luria Bertani (LB) broth medium (10 g/L peptone, 5 g/L yeast extract, and 20 g/L NaCl, pH adjusted to 7.0) and were incubated at 28°C, and the cultures were incubated to logarithmic phase with the optical density at 600 nm (OD_600_) reaching 0.8. The exponential-phase cells were added to LB agar medium (40°C–45°C) at a final concentration of 5 × 10^4^ cfu/mL. After solidification for 20 min, sterile filter paper impregnated with 3 μL of sample solution was placed on the plates and incubated at 28°C for 12 h. The anti-*Vibrio* effects were checked and recorded.

The minimal inhibitory concentration (MIC) assay of equisetin (**1**) and 5′-epiequisetin (**2**) toward these *Vibrio* strains was further determined with minor modification as described previously (Zhang et al., 2022). The *Vibrio* strains were cultivated in LB broth medium at 28°C, and the culture was incubated to exponential phase with the optical density at 600 nm (OD_600_) reaching 0.8. Briefly, the OD_600_ of exponential-phase cells of *Vibrio* was adjusted to 0.01 with LB broth medium. Thereafter, 150 μL of the *Vibrio* cells suspension was transferred into the wells of a 96-well microplate with different concentrations of **1** or **2**. DMSO (1%, v/v) was served as the negative control. The microplate was incubated at 28°C for 16 h and checked after incubation (Wiegand et al., 2008). The MIC values were defined as the lowest concentrations of **1** or **2** that inhibited the growth of *Vibrio* spp. (OD_600_ < 0.05).

Scanning electron microscopy (SEM) was further performed to investigate the morphological changes of *Vibrio* treated with compounds **1** and **2**. The strain *V. parahaemolyticus* was incubated on the LB as described above. Thereafter, compounds **1** and **2** (10 mg/mL) were added to the *Vibrio* cells suspension at a final concentration of 1.0 × MIC and incubated at 28°C for 12 h. The treated cells were collected for the SEM assay. The treated cells were washed three times by PBS, and fixed in 2.5% glutaraldehyde for 2 h. Then, the treated cells dehydrated successively in an ethanol series of 30%, 50%, 70%, 80%, 90%, and 100% tert-butyl alcohol for 10 min at each stage. The freeze-dried samples were analyzed with an SEM (Zeiss, Sigma 300) operated at 3 kV.

### Isolation and Structure Characterization of Equisetin **(1)** and 5′-Epiequisetin **(2)**

The fungal strain *F. equiseti* BBG10 was cultured on Müller Hinton broth (MB) agar plates (malt extract 15 g, artificial sea salt 15 g, and agar 20 g) at 25°C for 7 days. Then, it was inoculated in the seed medium (malt extract 15 g and artificial sea salt 15 g in 1.0-L tap distilled H_2_O, pH 7.4–7.8) at 25°C on a rotary platform shaker at 180 rpm for 48 h. Subsequently, a large-scale fermentation of *F. equiseti* BBG10 was carried out in modified rice solid medium (150 g rice, 3.0 g artificial sea salt, and 180 mL H_2_O) employing with 1 L × 20 Erlenmeyer flasks at room temperature for 30 days. The whole fermented cultures were extracted with EtOAc three times to provide a brown extract (50 g). The EtOAc crude extract was fractionated by medium pressure liquid chromatography (MPLC) using a step gradient elution with petroleum ether/CH_2_Cl_2_/MeOH (petroleum ether/CH_2_Cl_2_, 1:0–0:1; CH_2_Cl_2_/methanol, 1:0–1:1, v/v), which afforded 10 fractions (Frs.1 ~ 10) based on thin-layer chromatography (TLC) analysis. Fr.6 was further separated by semipreparative high performance liquid chromatography (HPLC) with MeCN/H_2_O (80:20, v/v, 5.0 mL/min) to yield compounds **1** (*t*_R_ = 38 min, 300 mg) and **2** (*t*_R_ = 43 min, 350 mg). Their structures were confirmed by high resolution-electron spray ionization mass spectrometry (HR-ESIMS) and high-performance liquid chromatography-diode array detection (HPLC-DAD) data analysis as well as comparison with the standard reference substances. HR-ESIMS spectra were collected on a Waters Xevo G2-S TOF mass spectrometer (Waters Corporation, United States).

## Results

### Identification and Phylogenetic Tree Analysis of Fungal Strains Derived From the Weizhou Islands Coral

**Figure 1:**
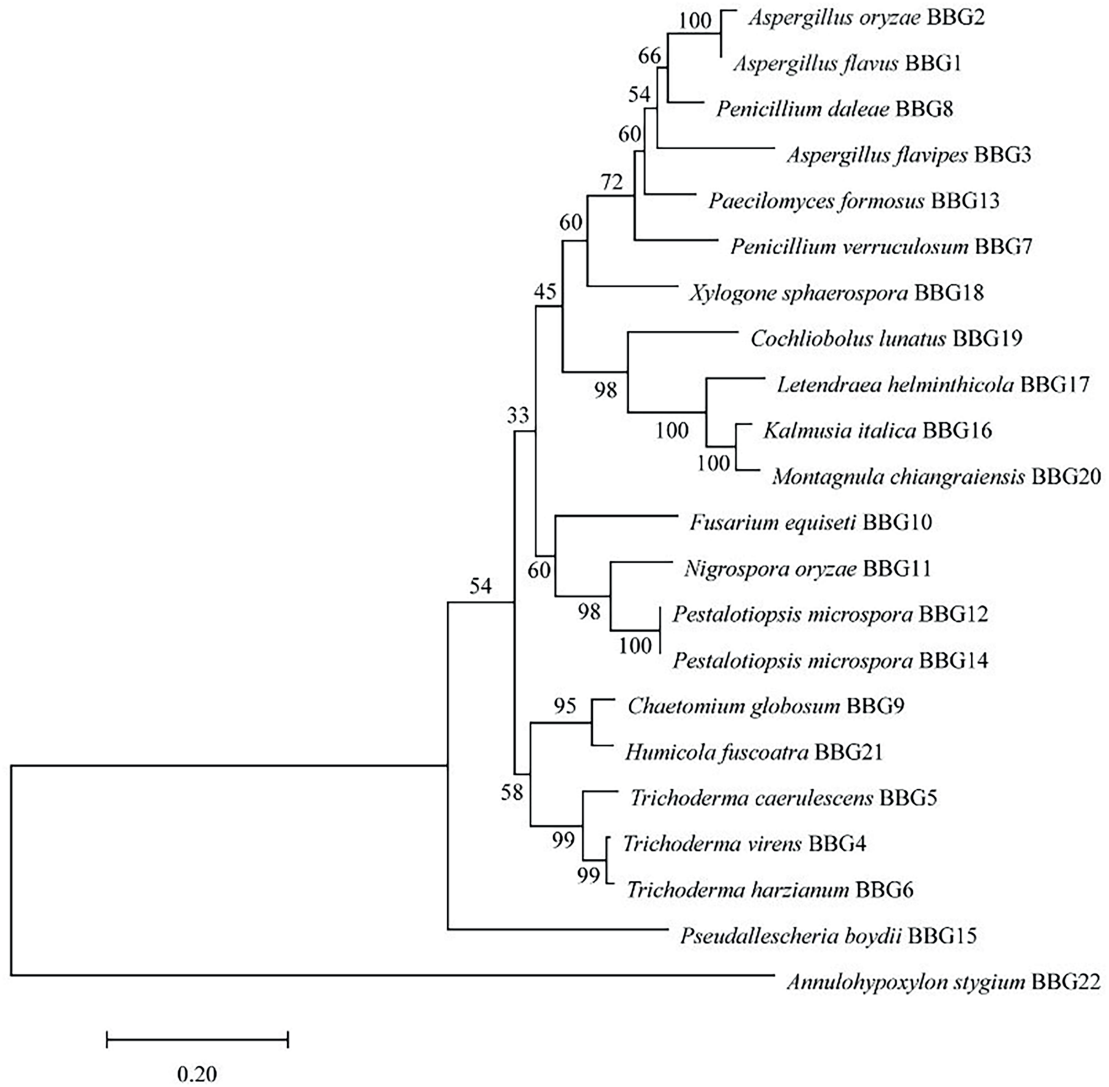
Phylogenetic tree analysis of 22 marine fungal strains.

Twenty-two candidate fungal strains were identified on the basis of the molecular protocol by amplification and sequencing of the DNA sequences of the ITS region of the rDNA gene. A classification of 22 strains based on the species name of the closely related species is shown in Figure 1. The predominant genera were *Aspergillus* and *Trichoderma*. Based on the ITS sequences, a phylogenetic tree was created using the neighbor-joining method to analyze the genetic relationship between 22 strains. The results showed that these strains could be divided into three major branches, while *Annulohypoxylon stygium* BBG22 is the most distant from other strains and belongs to a relatively independent branch. Moreover, the morphological property of the potential strain BBG10 was further collected to confirm the identification by using scanning electron microscopy (Supplementary Figure 1).

### Anti-*Vibrio* Activity of the Fungal Extracts

Cultivation of fungi from the Weizhou Islands coral yielded a total of 30 isolates. Reduplicate isolates were excluded under the guidance of observation of morphological differences, including the visible examination of growth characteristics, mycelia, and diffusible pigment. As a result, 22 independent strains (BBG1–BBG22) were selected for the screening of anti-bacterial activity against 6 strains of *Vibrio* spp. As shown in Table 1 and Supplementary Figure 2, the fungal extracts of 8 isolates (36.3%) displayed potential growth inhibition against *Vibrio* in the filter paper disk assay (75 μg extracts/per piece). It is worth noting that the fungal extracts of two isolates, *Trichoderma virens* BBG4 and *Trichoderma harzianum* BBG6, exhibited strong anti-*Vibrio* activity, and the sizes of the inhibition zone were larger than that of the positive control, chloramphenicol (150 μg/ml). A previous study reported that *T. virens* could produce gliotoxin with strong antimicrobial activity (Vargas et al., 2014). Besides, *T. harzianum* was reported to be a biocontrol bacterium for plant diseases (Altomare et al., 1999). The results also showed that different *Vibrio* species have divergent susceptibilities to extracts. Additionally, 22 fungal extracts were further analyzed by HPLC for the diversity of secondary metabolites. Therefore, *F. equiseti* BBG10 with interesting HPLC-DAD profiles (Supplementary Figure 3) of its crude extract was selected as the bioactive target strain to identify the active constituents.

**Table 1:** The anti-*Vibrio* activity of fungal extracts (diameter of inhibition zone, mm).

	*Vibrio alginalyticus*	*Vibrio coralliilyticus*	*Vibrio harveyi*	*Vibrio parahaemolyticus*	*Vibrio shilonii*	*Vibrio owensii*
Chl	1.69 ± 0.10	2.15 ± 0.22	2.00 ± 0.04	1.53 ± 0.07	2.01 ± 0.20	1.66 ± 0.20
*Aspergillus oryzae* BBG2	0.78 ± 0.06	0.93 ± 0.08	0	0.87 ± 0.19	0.90 ± 0.10	0.89 ± 0.09
*Aspergillus flavipes* BBG3	0	1.23 ± 0.21	0	0.75 ± 0.02	0.84 ± 0.07	0.72 ± 0.05
*T. virens* BBG4	1.73 ± 0.27	2.58 ± 0.03	1.96 ± 0.10	2.28 ± 0.07	2.62 ± 0.04	2.26 ± 0.36
*T. harzianum* BBG6	2.10 ± 0.25	2.20 ± 0.10	1.73 ± 0.15	2.36 ± 0.07	2.32 ± 0.12	1.19 ± 0.01
*Chaetomium globosum* BBG9	0.80 ± 0.04	1.64 ± 0.05	0.75 ± 0.03	0.81 ± 0.06	1.26 ± 0.07	1.33 ± 0.02
*F. equiseti* BBG10	0.80 ± 0.04	1.13 ± 0.08	0.71 ± 0.03	0.77 ± 0.03	0.94 ± 0.03	0.78 ± 0.10
*Nigrospora oryzae* BBG11	0.88 ± 0.07	1.02 ± 0.18	0.82 ± 0.12	0.85 ± 0.02	1.31 ± 0.06	1.28 ± 0.32
*A. stygium* BBG22	0	0	0.75 ± 0.06	0.85 ± 0.17	0.83 ± 0.06	0.92 ± 0.13

Chl, chloramphenicol.

### Production of Bioactive Metabolites

**Figure 2:**
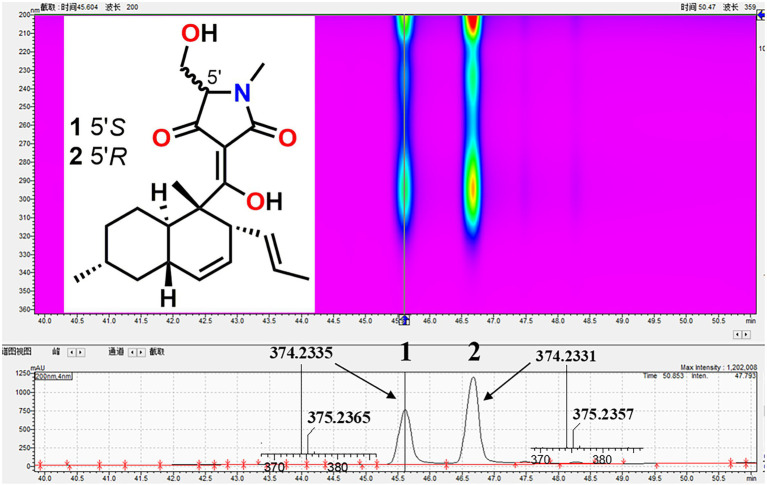
The isolation and structures of equisetin (**1**) and 5′-epiequisetin (**2**).

To investigate the bioactive constituents of *F. equiseti* BBG10, a large-scale fermentation was performed in 3 kg of rice solid medium. After harvest, its organic extract was further separated by MPLC and HPLC. Their structures were confirmed by HR-ESIMS and HPLC-DAD data analysis as well as comparison with the standard reference substances, which were identified as equisetin (**1**) and 5′-epiequisetin (**2**), respectively (Figure 2).

### Anti-*Vibrio* Activities of Compounds **1** and **2**

**Figure 3:**
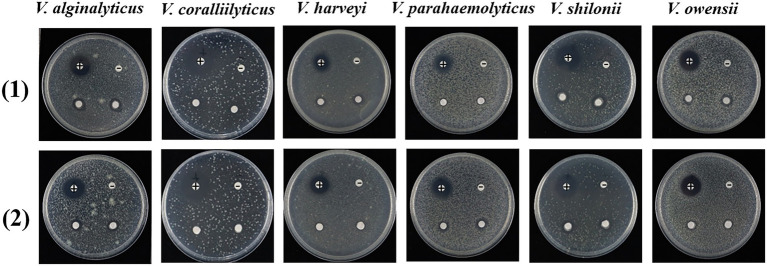
Inhibitory activities of **1** and **2** against *Vibrio*. Each plate contains four pieces of paper disk, positive control marks as “+,” negative control marks as “–,” and the other two pieces of paper disk contains compound.

Equisetin has been reported to have various biological actions, including antibacterial (Vesonder et al., 1979), anti-HIV (Hazuda et al., 1999; Clercq, 2000), antiobesity, and selective cytotoxicity to mammalian cells (Yin et al., 2013). However, this is the first report on the anti-*Vibrio* activity of equisetin (**1**) and 5′-epiequisetin (**2**) against *V. alginalyticus*, *V. coralliilyticus*, *V. harveyi*, *V. parahaemolyticus*, *V. owensii*, and *V. shilonii*. The filter paper disk assay showed that equisetin and 5′-epiequisetin exhibited weak bacteriostatic activity against *V. alginalyticus*, *V. parahaemolyticus*, *V. owensii*, and *V. shilonii* (Figure 3; Supplementary Table 1).

In addition, the MIC assay was further used to test the bacteriostatic ability of equisetin and 5′-epiequisetin. Both equisetin and 5′-epiequisetin showed inhibitory effects on six strains of *Vibrio* with the MIC values ranging from 86 to 132 μg/ml (Table 2). In order to clearly reflect the effect of compounds on the growth of tested bacteria, one of the Vibrio strain, *V. parahaemolyticus*, was selected to further investigate the growth curves of **1** and **2** at different concentrations (0.5 × MIC, 1.0 × MIC, and 2.0 × MIC), while the OD_600_ values were recorded within 16 h. As shown in Supplementary Figure 4, the growth of *V. parahaemolyticus* in the negative control and 0.5 × MIC (**1** and **2**) treatment groups entered the logarithmic growth period after 2 h, and the number of bacterial colonies kept growing within 16 h. Notably, the growth of *V. parahaemolyticus* was almost completely stagnant at the treatments of 1.0 × MIC and 2.0 × MIC, which suggested the bacteria were completely inhibited or even killed after treatment of compounds **1** and **2**. The above results indicated that anti-*Vibrio* effect of compounds **1** and **2** was in a dose-dependent manner.

**Table 2:** Anti-*Vibrio* activity of compounds **1** and **2** (MIC, μg/ml).

	*Vibrio alginalyticus*	*Vibrio coralliilyticus*	*Vibrio harveyi*	*Vibrio parahaemolyticus*	*Vibrio shilonii*	*Vibrio owensii*
**1**	119	119	132	119	119	119
**2**	86	106	106	106	106	86
Chl	1	1	1.2	1.2	1	1.2

Chl, chloramphenicol.

**Figure 4:**
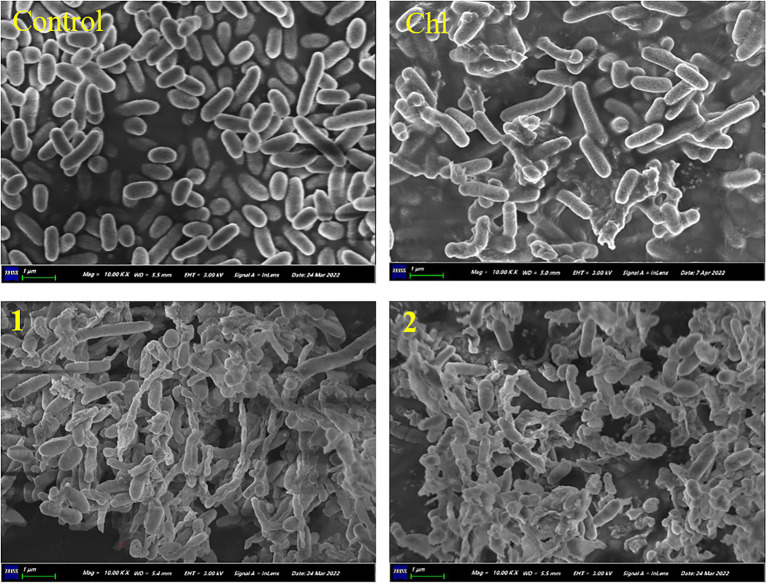
Electron microscopic observation of morphological changes in *Vibrio parahaemolyticus* cells following treatment with **1** and **2**. (Chl, chloramphenicol).

Thereafter, SEM was performed to investigate the morphological changes of *V. parahaemolyticus* treated with equisetin and 5′-epiequisetin. The results showed that the cell surfaces of the control group were smooth and that the *Vibrio* cells were plump and round. In contrast, the cell membranes became corrugated, distorted or ruptured after treatment with equisetin or 5′-epiequisetin. Interestingly, the destructive ability of equisetin and 5′-epiequisetin toward *Vibrio* cells were stronger than that of chloramphenicol. These results indicated that equisetin and 5′-epiequisetin can destroy the structure of *Vibrio* cell membranes (Figure 4).

## Discussion

The marine environment harbors a vast number of underexplored microbial resources. From a natural products perspective, marine microbes are better resources for novel anti-*Vibrio* lead compounds. Marine natural products represent a rich source of diverse molecules for drug development (Altmann, 2017). According to the Marinlit database, more than 36,000 compounds with diverse structures have been hitherto isolated from marine organisms, while over 1,000 new compounds have been isolated per year in the last decades. Notably, the proportion of novel compounds derived from marine microorganisms is gradually increasing (Carroll et al., 2022). To our knowledge, 15 marine drugs have been approved for marketing, including well-known cephalosporin and rifamycin from marine microorganisms. The Beibu Gulf is located in the tropics and subtropics, which is one of the regions with the most abundant microbial diversity in China. However, the research on microbial resources from the Beibu Gulf is relatively sparse. Therefore, it is promising to isolate and screen microbial resources and their bioactive metabolites from the Beibu Gulf.

*Vibrio* is a Gram-negative bacterium that is one of the main pathogenic bacteria of fish, shrimp, shellfish, and other marine animals (Letchumanan et al., 2015). Humans can also be infected by eating contaminated seafood, contact with seawater, etc. *Vibrio* pathogenicity mainly includes *V. parahaemolyticus*, *V. alginolyticus*, *V. vulnificus*, and *V. anguillarum* (Yu et al., 2012). The application of antibiotics is an effective method of *vibrio* control. There is an urgent need to find novel antibiotics against *Vibrio* (Preetha et al., 2015). In this work, we tried to screen new anti-*Vibrio* natural products from the fungal resources from the Beibu Gulf. We screened 22 fungal crude extracts, and eight fungal crude extracts showed different degrees of anti-*Vibrio* activity. Among them, the crude extracts of *T. virens* BBG4 and *T. harzianum* BBG6 exhibited particularly anti-*Vibrio* activity, and the size of the inhibition zone was larger than that of chloramphenicol. Two active components, equisetin and 5′-epiequisetin, were further identified from one of the bioactive strain, *F. equiseti* BBG10.

Equisetin and related derivatives have long been recognized for their wide biological activity against eukaryotic and bacterial cells, including antibacterial (Vesonder et al., 1979), anti-HIV (Hazuda et al., 1999; Clercq, 2000), anti-obesity, and selective cytotoxicity effects on mammalian cells (Yin et al., 2013). Previous reports indicate that equisetin functions in eukaryotic cells by affecting mitochondrial metabolism (Freiberg et al., 2004; Quek et al., 2013). Equisetin could affect malonyl-CoA synthesis as an acetyl-CoA carboxylase inhibitor (Freiberg et al., 2004; Larson et al., 2020). HIV integrase is inhibited by equisetin based upon its metal-binding property (Hazuda et al., 1999; Clercq, 2000).

In this work, equisetin and 5′-epiequisetin were identified from *F. equiseti* BBG10, and the crude extracts exhibited anti-*Vibrio* activity. The filter paper disk assay and MIC assay showed that equisetin and 5′-epiequisetin exhibited slight anti-*Vibrio* activity. Interestingly, the SEM results showed that the cell membranes became corrugated, distorted or ruptured after treatment with equisetin or 5′-epiequisetin. In contrast, the number of cells destroyed by chloramphenicol was less than those of equisetin and 5′-epiequisetin. These results indicated that equisetin and 5′-epiequisetin can more significantly damage the structure of *Vibrio* cell membranes than chloramphenicol, suggesting that the mechanism by which equisetin inhibits cell growth and kills cells is distinct from that of chloramphenicol. Moreover, *T. virens* BBG4 and *T. harzianum* BBG6 are potential strains for finding more potent anti-*Vibrio* compounds. This will be the focus of our future research.

## Conclusion

In summary, 22 fungal strains were isolated and identified from the Beibu Gulf coral *via* the serial dilution method and ITS sequence analysis, which were further divided into three branches by phylogenetic tree analysis, while eight fungal extracts were screened with potential anti-*Vibrio* activity *via* the filter paper disk assay. Further chemical investigation of the extracts of the target strain *F. equiseti* BBG10 *via* bioassay-guided isolation led to the characterization of two alkaloid-type antibiotics, equisetin and 5′-epiequisetin, which displayed anti-*Vibrio* activities against *V. alginalyticus*, *V. coralliilyticus*, *V. harveyi*, *V. parahaemolyticus*, *V. owensii*, and *V. shilonii*. Our research highlights the coral-derived microorganisms may be a large reservoir of bioactive natural products for future agrochemical development, and equisetin and 5′-epiequisetin could be promising lead compounds for the further development of novel anti-*Vibrio* agents.

## Data Availability Statement

The original contributions presented in the study are included in the article/Supplementary Material, further inquiries can be directed to the corresponding authors.

## Author Contributions

XL, BH, and YL conceived and designed the experiments, analyzed the data, wrote the manuscript, and prepared the figures and supplementary materials. XL, SP, and YZ isolated and purified fungi and compounds. XL, XX, and CG identified compounds. BH, SL, and YW performed anti-*Vibrio* assay and ITS sequencing. BH performed scanning electron microscopy. All authors commented on and approved the manuscript.

## Funding

This work was financially supported by the Specific Research Project of Guangxi for Research Bases and Talents (AD19110013), the Natural Science Foundation of Guangxi (2021GXNSF DA075010 and 2020GXNSFGA297002), the Guangxi Young and Middle-aged University Teachers’ Scientific Research Ability Enhancement Project (2021KY0315), the Special Fund for Bagui Scholars of Guangxi (YL), the National Natural Science Foundation of China (U20A20101), and the Scientific Research Foundation of Guangxi University of Chinese Medicine (2018006, 2018BS042, 2020QN025, 2019BS021, and 2018ZD005).

## Conflict of Interest

The authors declare that the research was conducted in the absence of any commercial or financial relationships that could be construed as a potential conflict of interest.

## Publisher’s Note

All claims expressed in this article are solely those of the authors and do not necessarily represent those of their affiliated organizations, or those of the publisher, the editors and the reviewers. Any product that may be evaluated in this article, or claim that may be made by its manufacturer, is not guaranteed or endorsed by the publisher.

## Supplementary Material

The Supplementary Material for this article can be found online at: https://www.frontiersin.org/articles/10.3389/fmicb. 2022.930981/full#supplementary-material
