# Supplementary material for: Isolation, Screening, and Active Metabolites Identification of Anti-Vibrio Fungal Strains Derived From the Beibu Gulf Coral
Source: Front Microbiol. 2022 Jun 2;13:930981. doi: 10.3389/fmicb.2022.930981 (PMC9201449; doi:10.3389/fmicb.2022.930981)
Supplement: Supplementary file 1 [file Data_Sheet_1.DOCX]

Supplementary Material

# Supplementary Figures and Tables

## Supplementary Figures


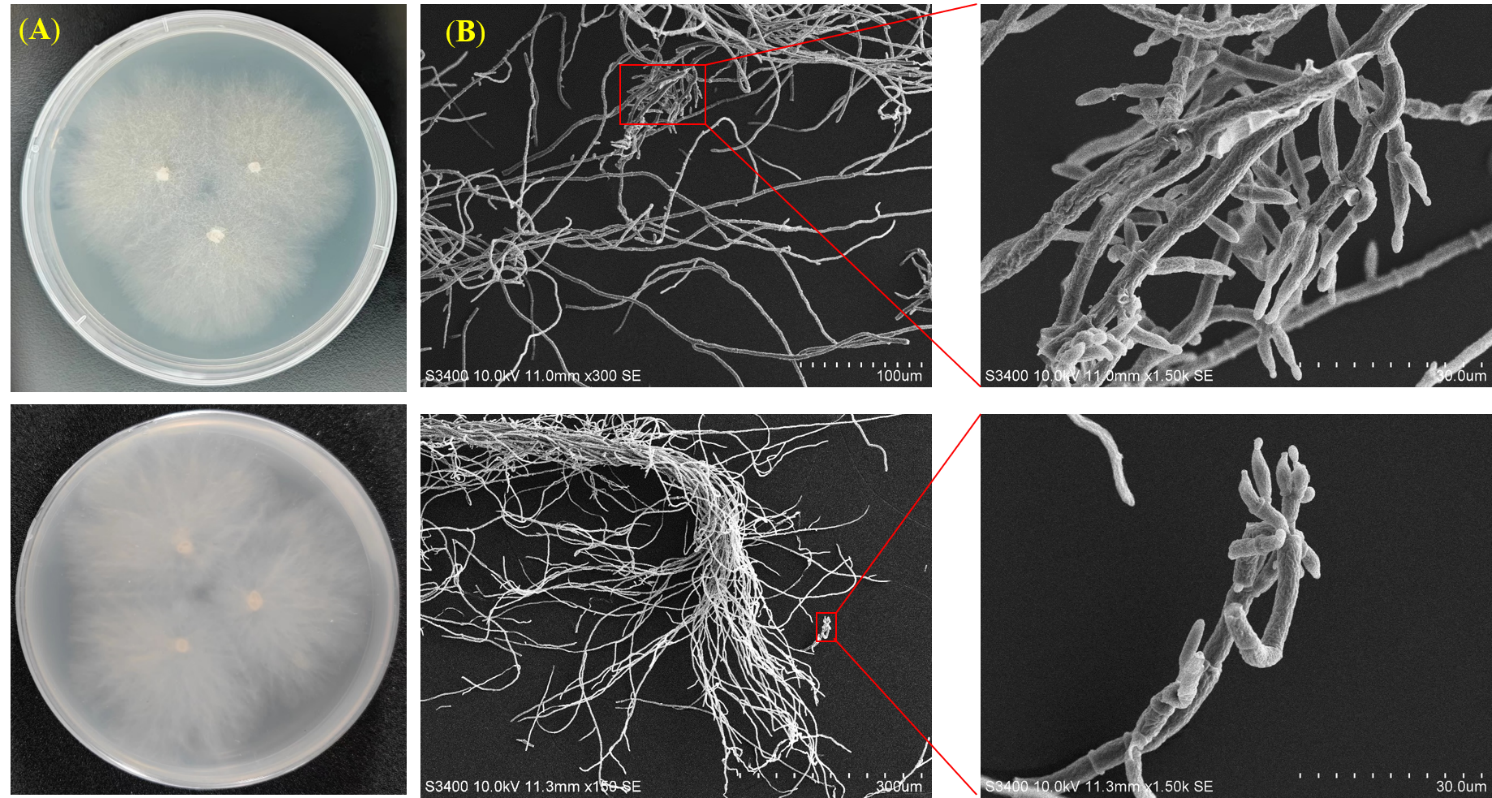


**Supplementary FIGURE 1** | Colony appearance and micromorphology of *F. equiseti* BBG10 (A). Electron microscopic observation of morphological characteristics of *F. equiseti* BBG10 (B).


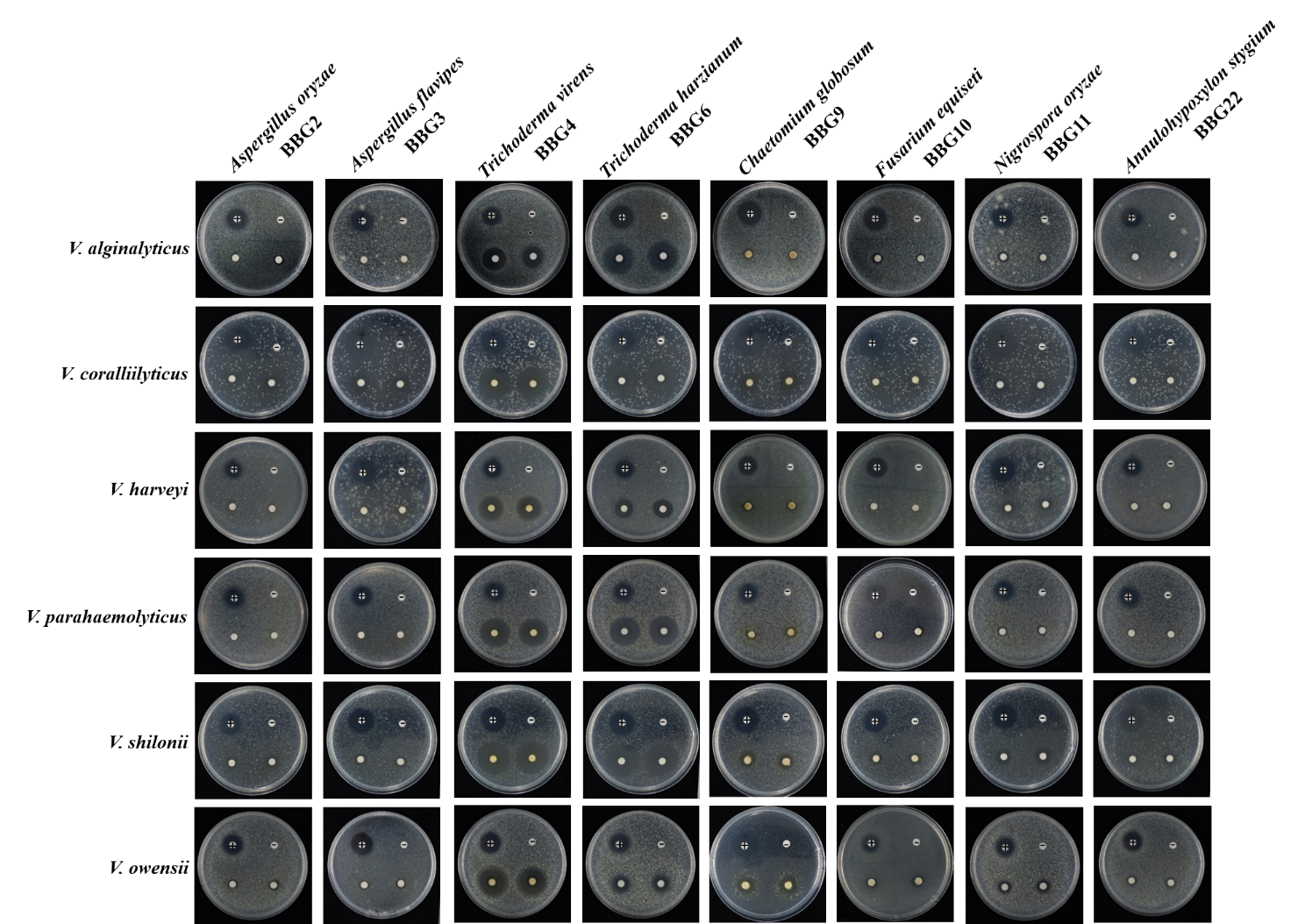


**Supplementary FIGURE 2** | Inhibitory activities of 8 fungal extracts against *Vibrio*. Each plate contains four pieces of paper disk, positive control marks as “+”, negative control marks as “-”, and the other two pieces of paper disk contains compound.


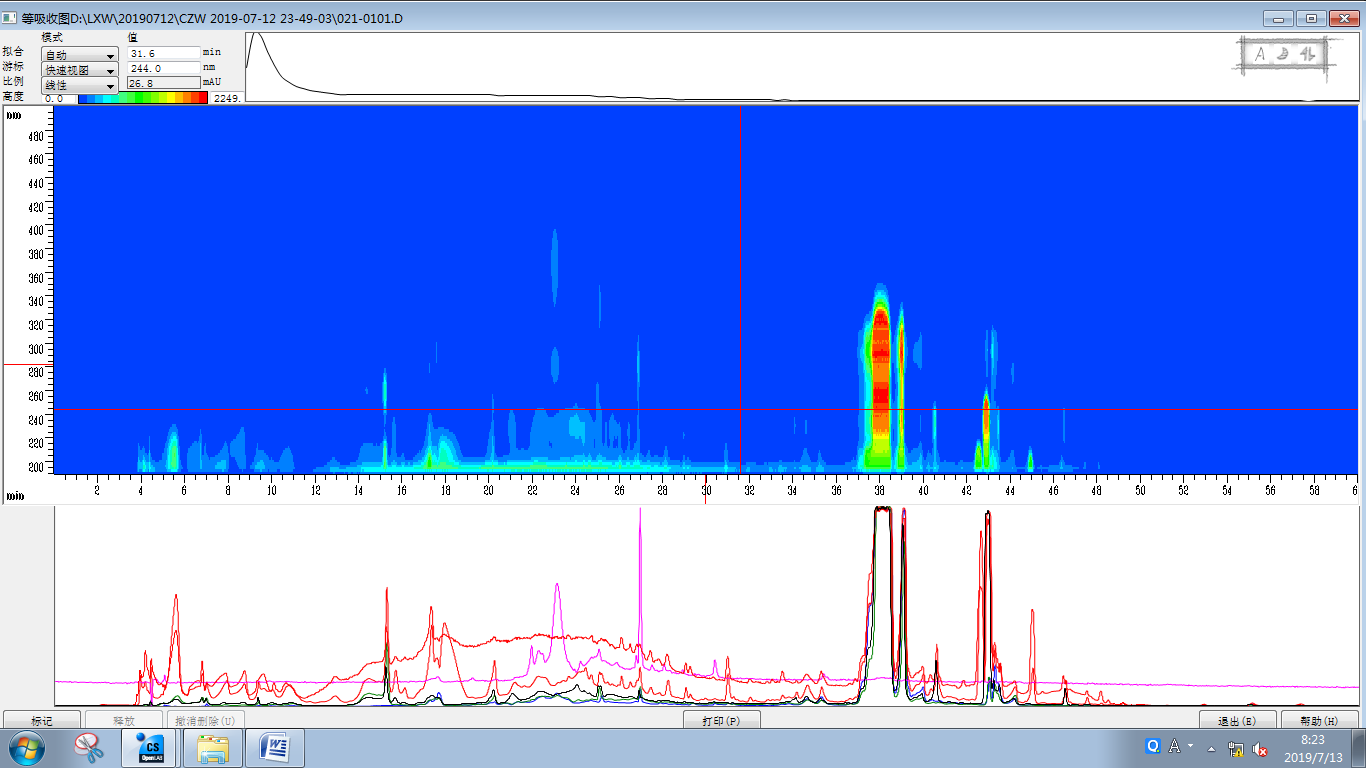


**Supplementary FIGURE 3** | HPLC-DAD profiles of the extracts of *F. equiseti* BBG10.


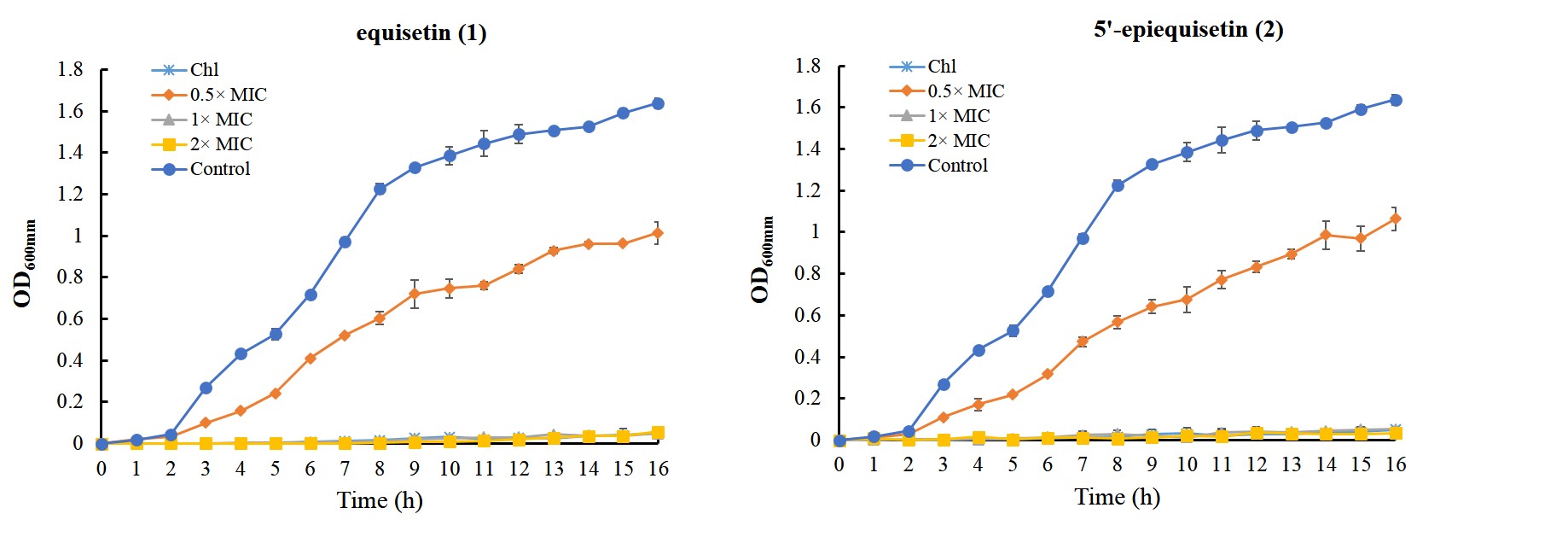


**Supplementary FIGURE 4** | Growth curves of *V. parahaemolyticus* treated by compounds **1** and **2**.

## Supplementary Tables

Supplementary TABLE 1 | The diameter of the inhibition zone of **1** and **2** (mm)

|  | *V.*  *alginalyticus* | *V.*  *coralliilyticus* | *V.*  *harveyi* | *V. parahae-molyticus* | *V.*  *shilonii* | *V.*  *owensii* |
| --- | --- | --- | --- | --- | --- | --- |
| **1** | 1.04±0.04 | 0 | 0.82±0.01 | 0.82±0.05 | 1.30±0.03 | 0.89±0.08 |
| **2** | 1.01±0.04 | 0 | 0.80±0.01 | 0.91±0.06 | 1.05±0.03 | 0.81±0.05 |
| Chl | 1.69±0.10 | 2.15±0.22 | 2.00±0.04 | 1.53±0.07 | 2.01±0.20 | 1.66±0.20 |

Chl: chloramphenicol.

# ITS sequences of strains BBG1-22

*Aspergillus flavus* BBG1 (GenBank accession no. ON459670):

CCTAGTCGGTCTAGCGAGCCACCTCCACCCGTGTTTACTGTACCTTAGTTGCTTCGGCGGGCCCGCCATTCATGGCCGCCGGGGGCTCTCAGCCCCGGGCCCGCGCCCGCCGGAGACACCACGAACTCTGTCTGATCTAGTGAAGTCTGAGTTGATTGTATCGCAATCAGTTAAAACTTTCAACAATGGATCTCTTGGTTCCGGCATCGATGAAGAACGCAGCGAAATGCGATAACTAGTGTGAATTGCAGAATTCCGTGAATCATCGAGTCTTTGAACGCACATTGCGCCCCCTGGTATTCCGGGGGGCATGCCTGTCCGAGCGTCATTGCTGCCCATCAAGCACGGCTTGTGTGTTGGGTCGTCGTCCCCTCTCCGGGGGGGACGGGCCCCAAAGGCAGCGGCGGCACCGCGTCCGATCCTCGAGCGTATGGGGCTTTGTCACCCGCTCTGTAGGCCCGGCCGGCGCTTGCCGAACGCAAATCAATCTTTTTCCAGGTTGACCTCGGATCAGGTAGGGATACCCGCTGAACTTAAGGATGACGATACTACGAGGAAAAAA

*Aspergillus oryzae* BBG2 (GenBank accession no. ON459671):

CACGAGGGAATTCGGGTCTAGCGAGCCACCTCCCACCCGTGTTTACTGTACCTTAGTTGCTTCGGCGGGCCCGCCATTCATGGCCGCCGGGGGCTCTCAGCCCCGGGCCCGCGCCCGCCGGAGACACCACGAACTCTGTCTGATCTAGTGAAGTCTGAGTTGATTGTATCGCAATCAGTTAAAACTTTCAACAATGGATCTCTTGGTTCCGGCATCGATGAAGAACGCAGCGAAATGCGATAACTAGTGTGAATTGCAGAATTCCGTGAATCATCGAGTCTTTGAACGCACATTGCGCCCCCTGGTATTCCGGGGGGCATGCCTGTCCGAGCGTCATTGCTGCCCATCAAGCACGGCTTGTGTGTTGGGTCGTCGTCCCCTCTCCGGGGGGGACGGGCCCCAAAGGCAGCGGCGGCACCGCGTCCGATCCTCGAGCGTATGGGGCTTTGTCACCCGCTCTGTAGGCCCGGCCGGCGCTTGCCAAACGCAAATCAATCTTTGTCCAGGTTGACCTCGGATCAGGGGGGGATACCCGGGGGACTTAAGCATATCATTCTCTTCCCAACCCCT

*Aspergillus flavipes* BBG3 (GenBank accession no. ON479332):

GTTGCATACCGACTGAGGTCTCGTGGCCCACCTCCCACCCGTGACTACTGTACCACTGTTGCTTCGGCGGGCCCGCCAGCCTAGCTGGCCGCCGGGGGGCTTCTGCCCCCGGGCCCGCCCCCTCCGGAGACCCCAACACCAACACTGTTTCTGAAAGCCTGTATGAATCCGATTCTTTGTAATCAGTTAAAACTTTCAACAATGGATCTCTTGGTTCCGGCATCGATGAAGAACGCAGCGAAATGCGATAACTAATGTGAATTGCAGAATTCAGTGAATCATCGAGTCTTTGAACGCACATTGCGCCCCCTGGTATTCCGGGGGGCATGCCTGTCCGAGCGTCATTACTGCCCTCAAGCCCGGCTTGTATTGGGTCCTCGTCCCCCCGGGGACGGGCCCAAAAGGCAGCGGCGGCACCGCGTCCGGTCCTCGAGCGTATGGGGCTTTGTCCCCCGCTGTGTGCGCGGGGCCGGCGACAGCCCACGCATATTTTCTTTTTTTTC

*Trichoderma virens* BBG4 (GenBank accession no. ON459690):

CGCTATCACTCAACCCATGTGACGTTACCAAACTGTTGCCTCGGCGGGATCTCTGCCCCGGGTGCGTCGCAGCCCCGGACCAAGGCGCCCGCCGGAGGACCAACCAAAACTCTTATTGTATACCCCCTCGCGGGTTTTTTTACTATCTGAGCCATCTCGGCGCCCCTCGTGGGCGTTTCGAAAATGAATCAAAACTTTCAACAACGGATCTCTTGGTTCTGGCATCGATGAAGAACGCAGCGAAATGCGATAAGTAATGTGAATTGCAGAATTCAGTGAATCATCGAATCTTTGAACGCACATTGCGCCCGCCAGTATTCTGGCGGGCATGCCTGTCCGAGCGTCATTTCAACCCTCGAACCCCTCCGGGGGGTCGGCGTTGGGGATCGGCCCTTTACGGGGCCGGCCCCGAAATACAGTGGCGGTCTCGCCGCAGCCTCTCCTGCGCAGTAGTTTGCACACTCGCATCGGGAGCGCGGCGCGTCCACAGCCGTTAAACACCCCAAACTTCTGAAATGTTGACCTCGGATCAGGTAGGAATACCCGCTGAACTTAATCATACTCAAAAAAG

*Trichoderma caerulescens* BBG5 (GenBank accession no. ON459689):

GCGATAACTCCAACCAATGTGACGTTACCAAACTGTTGCCTCGGCGGGGTCACGCCCCGGGTGCGTAAAAGCCCCGGAACCAGGCGCCCGCCGGAGGGACCAACCAAACTCTTTTCTGTAGCCTTCGGGCGTTATTTCTTACAGCTCTGAGCAAAAATTCAAAATGAATCAAAACTTTCAACAACGGATCTCTTGGTTCTGGCATCGATGAAGAACGCAGCGAAATGCGATAAGTAATGTGAATTGCAGAATTCAGTGAATCATCGAATCTTTGAACGCACATTGCGCCCGCCAGTATTCTGGCGGGCATGCCTGTCCGAGCGTCATTTCAACCCTCGAACCCCCTAGGGGGTCCGGCGTTGGGGATCGGGAACCCCTAAGACGGGATCCCGGCCCCGAAATACAGTGGCGGTCTCGCCGCAGCCTCTCCTGCGCAGTAGTTTGCACAACTCGCACCGGGAGCGCGGCGCGTCCACGTCCGTAAAACACCCAACTTCTGAAATGTTGACCTCGGATCAGTAGGAATACCCGCTGAACTT

*Trichoderma harzianum* BBG6 (GenBank accession no. ON459695):

GCTTATCGTACTCCAACCATGTGACGTTACCAAACTGTTGCCTCCGGCGGGATCTCTGCCCCGGGTGCGTCGCAGCCCCGGACCAAGGCGCCCGCCGGAGGACCAACCAAAACTCTTATTGTATACCCCCTCGCGGGTTTTTTTATAATCTGAGCCTTCTCGGCGCCTCTCGTAGGCGTTTCGAAAATGAATCAAAACTTTCAACAACGGATCTCTTGGTTCTGGCATCGATGAAGAACGCAGCGAAATGCGATAAGTAATGTGAATTGCAGAATTCAGTGAATCATCGAATCTTTGAACGCACATTGCGCCCGCCAGTATTCTGGCGGGCATGCCTGTCCGAGCGTCATTTCAACCCTCGAACCCCTCCGGGGGGTCGGCGTTGGGGATCGGCCCTGCCTCTTGGCGGCGGCCGTCTCCGAAATACAGTGGCGGTCTCGCCGCAGCCTCTCCTGCGCAGTAGTTTGCACACTCGCATCGGGAGCGCGGCGCGTCCACAGCCGTTAAACACCCAACTTTCTGAAATGTTGACCGCGGATCAGGTAGGAATACCCGCTGAACTTAAGGCGGGCTTCTTAGGGCGGAGGAA

*Penicillium verruculosum* BBG7 (GenBank accession no. ON459694):

TCCTGAAGGGGGCTCGCGGCTCACCTCCACCCTTGTCTCTCTACACCCTGTTGCTTTGGCGGGCCCACCGGGGCCACCCGGTCGCCGGGGGACGCACGTCCCCGGGCCCGCGCCCGCCGAAGCGCTCTGTGAACCCTGATGAAGATGGGCTGTCTGAGTACTATGAAAATTGTCAAAACTTTCAACAATGGATCTCTTGGTTCCGGCATCGATGAAGAACGCAGCGAAATGCGATAAGTAATGTGAATTGCAGAATTCCGTGAATCATCGAATCTTTGAACGCACATTGCGCCCCCTGGCATTCCGGGGGGCATGCCTGTCCGAGCGTCATTTCTGCCCTCAAGCACGGCTTGTGTGTTGGGTGCGGTCCCCCCGGGGACCCGCCCGAAAGGCAGCGGCGACGTCCGTCTGGTCCTCGAGCGTATGGGGCTTTGTCACTCGCTCGGGAAGGACCTGCGGGGGTTGGTCACCACCATATTTTTACCACGGTTGACCTCGGATCAGGTAGGAGTTACCCGCTGAACTTAAGCATATC

*Penicillium daleae* BBG8 (GenBank accession no. ON459700):

GTGCCGTCTGTGAAGGCCTCTGGGTCCACCTCCCACCCGTGTTTATCGTACCTTGTTGCTTCGGCGGGCCCGCCTCACGGCCGCCGGGGGGCATCTGCCCCCGGGCCCGCGCCCGCCGAAGACACCATTGAACGCTGTCTGAAGATTGCAGTCTGAGCATCTTAGCTAAATCAGTTAAAACTTTCAACAACGGATCTCTTGGTTCCGGCATCGATGAAGAACGCAGCGAAATGCGATAAGTAATGTGAATTGCAGAATTCAGTGAATCATCGAGTCTTTGAACGCACATTGCGCCCTCTGGTATTCCGGAGGGCATGCCTGTCCGAGCGTCATTGCTGCCCTCAAGCACGGCTTGTGTGTTGGGTCCCCGCCCCCTGCTTCCAGGGGGCGGACCCGAAAGGCAGCGGCGGCACCGCGTCCGGTCCTCGAGCGTATGGGGCTTCGTCACCCGCTCTTGTAGGCCCGGCCGGCGCCCGCCGGCGACCCCCCATCAATCTTTTCAGGTTGACCTCGGATCAGGTAGGGATACCCGCTGAACTTAAGCATA

*Chaetomium globosum* BBG9 (GenBank accession no. ON459772):

AAGGTACAGGCAACTCCCTAACCATTGTGACGTTACCTATACCGTTGCTTCGGCGGGCGGCCCCGGGGTTTACCCCCCGGGCGCCCCTGGGCCCCACCGCGGGCGCCCGCCGGAGGTCACCAAACTCTTGATAATTTATGGCCTCTCTGAGTCTTCTGTACTGAATAAGTCAAAACTTTCAACAACGGATCTCTTGGTTCTGGCATCGATGAAGAACGCAGCGAAATGCGATAAGTAATGTGAATTGCAGAATTCAGTGAATCATCGAATCTTTGAACGCACATTGCGCCCGCCAGCATTCTGGCGGGCATGCCTGTTCGAGCGTCATTTCAACCATCAAGCCCCCGGGCTTGTGTTGGGGACCTGCGGCTGCCGCAGGCCCTGAAAAGCAGTGGCGGGCTCGCTGTCGCACCGAGCGTAGTAGCATACATCTCGCTCTGGTCGCGCCGCGGGTTCCGGCCGTTAAACCACCTTTTAACCCAAGGTTGACCTCGGATCAGGTAGGAAACCCGCTGAACTTAGGGGTATACTTCAAAGCGGAAAGAATG

*Fusarium equiseti* BBG10 (GenBank accession no. ON459771):

ACTTTAATCCTCCAACCCTGTGACATACCTATACGTATGCCTCCGGCGGATCAGCCCGCGCCCCGTAAAACGGGACGGCCCGCCCGAGGACCCCTAAACTCTGTTTTTAGTGGAACTTCTGAGTAAAACAAACAAATAAATCAAAACTTTCAACAACGGATCTCTTGGTTCTGGCATCGATGAAGAACGCAGCAAAATGCGATAAGTAATGTGAATTGCAGAATTCAGTGAATCATCGAATCTTTGAACGCACATTGCGCCCGCCAGTATTCTGGCGGGCATGCCTGTTCGAGCGTCATTTCAACCCTCAAGCTCAGCTTGGTGTTGGGACTCGCGGTAACCCGCGTTCCCCAAATCGATTGGCGGTCACGTCGAGCTTCCATAGCGTAGTAATCATACACCTCGTTACTGGTAATCGTCGCGGCCACGCCGTTAAACCCCAACTTCTGAATGTTGACCTCGGATCAGGTAGGAATACCCGCTGAACTTAAGCATATCAATAAAAACGGAGAGAA

*Nigrospora oryzae* BBG11 (GenBank accession no. ON459773):

ACGTATTCACTCCAACCATGTGACATATCTCTTTGTTGCCTCGGCGCAAGCTACCCGGGACCTCGCGCCCCGGGCGGCCCGCCGGCGGACAAACCAAACTCTGTTATCTTCGTTGATTATCTGAGTGTCTTATTTAATAAGTCAAAACTTTCAACAACGGATCTCTTGGTTCTGGCATCGATGAAGAACGCAGCGAAATGCGATAAGTAATGTGAATTGCAGAATTCAGTGAATCATCGAATCTTTGAACGCACATTGCGCCCATTAGTATTCTAGTGGGCATGCCTGTTCGAGCGTCATTTCAACCCCTAAGCACAGCTTATTGTTGGGCGTCTACGTCTGTAGTGCCTCAAAGACATTGGCGGAGCGGCAGCAGTCCTCTGAGCGTAGTAATTCTTTATCTCGCTTTTGTTAGGCGCTGCCCCCCCGGCCGTAAAACCCCCAATTTTTTCTGGTTGACCTCGGATCAGGTAGGAATACCCGCTGAACTTAAGCATATCGAGAGATCGGGGGGAAG

*Pestalotiopsis microspora* BBG12 (GenBank accession no. ON459774):

AGAATATCTACTCCACCCATGTGACTTACCATTGTTGCCTCGGCAGAAGCTGCTCGGCGCGCCTTACCTTGGAACGGCCTACCCTGTAGCGCCTTACCCTGGAACGGCTTACCCTGCAACGGCTGCCGGTGGACTACCAAACTCTTGTTATTTTATGGTTATCTGAGCGTCTTATTTTAATAAGTCAAAACTTTCAACAACGGATCTCTTGGTTCTGGCATCGATGAAGAACGCAGCGAAATGCGATAAGTAATGTGAATTGCAGAATTCAGTGAATCATCGAATCTTTGAACGCACATTGCGCCCATTAGTATTCTAGTGGGCATGCCTGTTCGAGCGTCATTTCAACCCTTAAGCCTAGCTTAGTGTTGGGAGCCTACTGCTTTTGCTAGCTGTAGCTCCTGAAATACAACGGCGGATCTGCGATATCCTCTGAGCGTAGTAATTTTTATCTCGCTTTTGACTGGAGTTGCAGCGTCTTTAGCCGCTAAACCCCCCAATTTTTAATGGTTGACCTCGGATCAGGTAGGAATACCCGCTGAACTTAAGCATATCAATA

*Paecilomyces formosus* BBG13 (GenBank accession no. ON459775):

TTCTTGAGGAAGGGTCACGAGCCCACCTCCATCCGTGTTGACTACACCTGTTGCTTCGGCGGGCCCGCCGTGGTTCACGCCCGGCCGCCGGGGGGCCTTGTGCTCCCGGGCCCGCGCCCGCCGAAGACCCCTCGAACGCTGCCCTGAAGGTTGCCGTCTGAGTATAAAATCAATCATTAAAACTTTCAACAACGGATCTCTTGGTTCCGGCATCGATGAAGAACGCAGCGAAATGCGATAAGTAATGTGAATTGCAGAATTCCGTGAATCATCGAATCTTTGAACGCACATTGCGCCCCCTGGCATTCCGGGGGGCATGCCTGTCCGAGCGTCATTGCTAACCCTCCAGCCCGGCTGGTGTGTTGGGTCGACGTCCCCCCCGGGGGACGGGCCCGAAAGGCAGCGGCGGCGCCGCGTCCGATCCTCGAGCGTATGGGGCTTTGTCACGCGCTCTGGTAGGGTCGGCCGGCTGGCCAGCCAGCGACCTCACGGTCACCTATTTTTTCTCTTAGGTTGACCTCGGATCAGGTAGGGATACCCGCTGAACTTAAGCAT

*Pestalotiopsis microspore* BBG14 (GenBank accession no. ON460248):

CCGTCATTCTACTCCACCCATGTGACTTACCATTGTTGCCTCGGCAGAAGCTGCTTGGCGCGCCTTACCTTGGAACGGCCTACCCTGTAGCGCCTTACCCTGGAACGGCTTACCCTGCAACGGCTGCCGGTGGACTACCAAACTCTTGTTATTTTATGGTTATCTGAGCGTCTTATTTTAATAAGTCAAAACTTTCAACAACGGATCTCTTGGTTCTGGCATCGATGAAGAACGCAGCGAAATGCGATAAGTAATGTGAATTGCAGAATTCAGTGAATCATCGAATCTTTGAACGCACATTGCGCCCATTAGTATTCTAGTGGGCATGCCTGTTCGAGCGTCATTTCAACCCTTAAGCCTAGCTTAGTGTTGGGAGCCTACTGCTTTTGCTAGCTGTAGCTCCTGAAATACAACGGCGGATCTGCGATATCCTCTGAGCGTAGTAATTTTTATCTCGCTTTTGACTGGAGTTGCAGCGTCTTTAGCCGCTAAACCCCCCAATTTTTAATGGTTGACCTCGGATCAGGTAGGAATACCCGCTGAACTTAAGCATATCAAT

*Pseudallescheria boydii* BBG15 (GenBank accession no. ON460249):

ACCTAGTCTCTCAAACCATTGTGACTTACCTATGTTCTGTTGCCTCGGCGGCGTGGTCAGCGCCCCCTCTGAAAAGAGGACGATGCCCTCCCGCCGGCAGCACCAAACTCTTGAATTTTACAGCGGATTACAGTTCTGATTTGAAAAGAAAAAACAAGTTAAAACTTTCAACAACGGATCTCTTGGTTCTGGCATCGATGAAGAACGCAGCGAAATGCGATAAGTAATGTGAATTGCAGAATTCAGTGAATCATCGAATCTTTGAACGCACATTGCGCCCGGCAGTAATCTGCCGGGCATGCCTGTCCGAGCGTCATTTCAACCCTCGAACCTCCGTTTCCTCAGGGAAGCTCAGGGTCGGTGTTGGGGCGCTACGGCGAGTCTTCGCGACCCTCCGTAGGCCCTGAAATACAGTGGCGGTCCCGCCGCGGTTGCCTTCTGCGTAGTAAGTCTCTTTTGCAAGCTCGCATTGGGTCCCGGCGGAGGCCTGCCGTCAAACCACCTATAACTCCAGATGGTTTGACCTCGGATCAGGTAGGGTTACCCGCTGAACTTAACCA

*Kalmusia italica* BBG16 (GenBank accession no. ON460250):

GGTTTTAGGCGTACAGCCTTACCCTTCTCTACACGTACCTTCACATTCTCCTTCGGCGGGGTAACGCCCGCCGTCGGAACCATCCAAACCCTTTTTTTTGCATCTAGCATCCTACCCGTTCTGATACAAAATTCAATCGTTACAACTTTCAACAATGGATCTCTTGGCTCTGGCATCGATGAAGAACGCAGCGAAATGCGATAAGTAGTGTGAATTGCAGAATTCAGTGAATCATCGAATCTTTGAACGCACATTGCGCCCCTTGGTATTCCATGGGGCATGCCTGTTCGAGCGTCATCTACACCCTCAAGCTCTGCTTGGTGTTGGGCGTCTGTCCCGCCCCCGCGCGTGGACTCGCCCCAAATCCATTGGCAGCGTTCCTTGCCCCCTCTCGCGCAGCACATTGCGCTTCTCGAGGGCGGCTCCGGGACGCGATCCAGCAAGATGACCACCGTCTTTGACCTCGGATCAGGTAGGGATACCCGCTGAACTTAAGCATATAAAAAAACTAAAGAGGAAA

*Letendraea helminthicola* BBG17 (GenBank accession no. ON460251):

GTTCTTTGGTTTCACACGATGGCGTCGTCCTTAGAACCGTCTCCGTGCGGCTCGGGGCGGCGTTTCATCAGCGGGCACGTCGCGGCTTCCTCTCAGGAAGTAATCACGCGGGGTCGTCTGAATCCTTAACTTTACGAGAACTCCCCATACTCCTTCGGTGGGGTGACCTGCCGTTGGAACCAACAAAAAACTTTTTTTGCATCTAGTATTACCTGTTCTGATACAAACAATCGTTACAACTTTCAACAATGGATCTCTTGGCTCTGGCATCGATGAAGAACGCAGCGAAATGCGATAAGTAGTGTGAATTGCAGAATTCAGTGAATCATCGAATCTTTGAACGCACATTGCGCCCCTTGGTATTCCATGGGGCATGCCTGTTCGAGCGTCATCTACACCCTCAAGCTCTGCTTGGTGTTGGGCGTCTGTCCCGCCTCCGCGCGTGGACTCGCCCCAAATTCATTGGCAGCGGTCTTCTTGCCCCCTCTCGTGCAGCACATTGCGCTTCTCGAGGGCAGCGGGCCGCGTCCACGAAGCAACATTCACCGTCGTTGACCTCGGATCAGGTAGGGATACCCGCTGAGGTTGAGCATAACGACCTCCTATGA

*Xylogone sphaerospora* BBG18 (GenBank accession no. ON460252):

GTGTCTTCCAGTCGTGCCTCACGGGTAGATCTCCCACCCTTGTGTATTCTCTACATCTTGTTGCTTTGGCAGGCCGTTGGTTCGCCAACCACCGGCTCGCGGGCTGGTGCGTGCCTGCCAGAGGACCTCTCAAACTCGGTTTGTCAGTGTCGTCTGAGTACCATAACAATCGTTAAAACTTTCAACAACGGATCTCTTGGTTCTGGCATCGATGAAGAACGCAGCGAAATGCGATAAGTAATGTGAATTGCAGAATTCAGTGAATCATCGAATCTTTGAACGCACATTGCGCCCCTTGGTATTCCGAGGGGCATGCCTGTTCGAGCGTCATTTCAACCCCTCAAGCTTAGCTTGGTGTTGGGCTGCGCCAGCGTTGCTGGCGGGCCTTAAAATCAGTGGCGGTGCCGTTTGGGCTCCAAGCGTAGTAGCATCTCTCGCTCTGGAGACCCGGCGGTTGCTTGCCAGACAATCACTAAAAAAACAAAGGTTTGACCTCGGATCAGGTAGGGATACCCGCTGAACTTAAGCATATCAGACGGACAGAGAGAAA

*Cochliobolus lunatus* BBG19 (GenBank accession no. ON460257):

TCGGACTTATAATATGAGGCTGTACGCGGCTGTGCTCTCGGGCCAGTTTTGCGGAGGCTGAATTATTTATTACCCTTGTCTTTTGCGCACTTGTTGTTTCCTGGGCGGGTTCGCCCGCCACCAGGACCACATCATAAACCTTTTTTATGCAGTTGCAATCAGCGTCAGTATAACAAATGTAAATCATTTACAACTTTCAACAACGGATCTCTTGGTTCTGGCATCGATGAAGAACGCAGCGAAATGCGATACGTAGTGTGAATTGCAGAATTCAGTGAATCATCGAATCTTTGAACGCACATTGCGCCCTTTGGTATTCCAAAGGGCATGCCTGTTCGAGCGTCATTTGTACCCTCAAGCTTTGCTTGGTGTTGGGCGTTTTTTGTCTTTGGTTGCCAAAGACTCGCCTTAAAAGGATTGGCAGCCGGCCTACTGGTTTCGCAGCGCAGCACATTTTTGCGCTTGCAATCAGCAAAAGAGGACGGCAATCCATCAAGACTCCTTCTCACGTTTGACCTCGGATCAGGTAGGGATACCCGCTGAACTTAAGCATATCAATAAATGAGAGGAAAAGG

*Montagnula chiangraiensis* BBG20 (GenBank accession no. ON460258):

GGGTATTCTTTTGGCGTACAGCCTTAACCTTATCTATGCGTACCTTCACATTCTCCTTCGGCGGGGTAACGCCCGCCGTCGGAACAACAAACCCATTTGCATCTAGTATTCTACCAGTTCTGATAAAAATTCAATCGTTACAACTTTCAACAATGGATCTCTTGGCTCTGGCATCGATGAAGAACGCAGCGAAATGCGATAAGTAGTGTGAATTGCAGAATTCAGTGAATCATCGAATCTTTGAACGCACATTGCGCCCCTTGGTATTCCATGGGGCATGCCTGTTCGAGCGTCATCTACACCCTCAAGCTCTGCTTGGTGTTGGGCGTCTGTCCCGCCTCCGCGCGCGGACTCGCCCCAAATCCATTGGCAGCGGTCCTTGCCCCCTCTCGCGCAGCACATTGCGCTCCTCGAGGGCGGCTCCGGGCCTGCGACCCACGAAGATGACCCGTCTTTTGACCTCGGATCAGGTAGGGATACCCGCTGAACTTAAGCATATCAATGAGCGGGAGGAAG

*Humicola fuscoatra* BBG21 (GenBank accession no. ON460259):

GTGGAACGAGAGTTGCAACTCCCTAAACCATTGTGAACGTTACCTAAACCGTTGCTTCGGCGGGCGGCCCGGGTCCTTCCCGGCGCCCCTCGGCCCTCGCGGGCGCCCGCCGGAGGTAAACCAAACTATTGCATTGTATGGCCTCTCTGAGTCTTCTGTACTGAATAAGTCAAAACTTTCAACAACGGATCTCTTGGTTCTGGCATCGATGAAGAACGCAGCGAAATGCGATAAGTAATGTGAATTGCAGAATTCAGTGAATCATCGAATCTTTGAACGCACATTGCGCCCGCCAGTATTCTGGCGGGCATGCCTGTTCGAGCGTCATTTCAACCATCAAGCCCCGGGCTTGTGTTGGGGACCTGCGGCTGCCGCAGGCCCTGAAATGCAGTGGCGGGCTCGCTGTCACTCCGAGCGTAGTAGTTACATCTCGCTCTGGGCGTGCTGCGGGTTCCGGCCGTTAAAAGCCTTATTTACCCAAGGTTGACCTCGGATCAGGTAGGAAGACCCGCTGAACTTAAGCATGTATTGTAAGCGGAAGAA

*Annulohypoxylon stygium* BBG22 (GenBank accession no. ON479614):

ACGTGTCTCTGGTACAAACTCCACCCTTTGTGACCTACCTATGTTTCCTCCGGCGTACCGCTTTAGCCTACCCACAGGGCTCCCCTAAGGGGGGGTTCTGCTGGGGAGGTGCCTGAGTGCTACCTATCCTTCGGGGTACGGTTAGTGCAGTGAAGGTGCTGACCAAGGCCTCGGCGGCGCCGAGTAGGACCGCTCCAAACTTAAGCACCTAGTGCATCCAACCCCGCGTTGAACAACTATCGAAAATCTGCTTTTGCTTTTTTTCTTTACGCTAAAACGTCTTTCCCGGTTGGAATTATTGCTCGAAATAATAATTTCTTTACCCTGCAGTCGTTTGTTTTCAAGCTACAATATCTGCTCGAAAATTGTTCAAAGCTCTGAGGGGTCTGAATGAATTCATAAAATTGGCAAAAGCCACCTATAAACTACGGTTCTTAGGGGGTGATCAAACCAAGGTTTTAAAAACCAAATACGTTAAAACTTTCAACAACGGATCTCTTGGTTCTGGCATCGATGAAGAACGCAGCGAAATGCGATAAGTAATGTGAATTGCAGAATTCAGTGAATCATCGAATCTTTGAACGCACATTGCGCCCATTAGTATTCTAGTGGGCATGCC

# Spectral data of compounds 1 and 2.

Equisetin (**1**)：^1^H NMR(500 MHz, CD_3_OD) *δ*_H_: 5.36~5.43(2H, m, H-4/5), 5.16~5.29 (2H, m, H-13/14), 3.96 (1H, dd, *J* = 7.0, 3.5 Hz, H-6'a), 3.89 (2H, qd, *J* = 7.0, 3.5 Hz, H-6'b), 3.68 (1H, br s, H-5'), 3.35 (1H, m, H-3), 3.03 (3H, s, H_3_-7'), 2.00 (1H, br s, H-10a), 1.85~1.70 (4H, m, H_2_-7, H_2_-9), 1.52 (3H, d, *J* =6.3 Hz, H_3_-15), 1.46 (3H, s, H_3_-12), 1.10 (2H, m, H-6, H-10b), 0.94 (3H, d, *J* = 6.5 Hz, H-16), 0.86 (1H, m, H-11); ^13^C NMR(125 MHz, CD_3_OD) *δ*_C_: 198.7(C, C-4'), 192.5 (C, C-1), 176.5 (C, C-2'), 132.4 (CH, C-5/C-13), 131.2 (CH, C-5/C-13), 127.8 (CH, C-4/C-14), 103.8 (C, C-3'), 69.4 (CH, C-5'), 59.8 (CH_2_, C-6'), 49.4 (C, C-2), 46.5 (CH, C-3), 43.6 (CH_2_, C-7), 41.4 (CH, C-11), 40.0 (CH, C-6), 37.0 (CH_2_, C-9), 34.8 (CH, C-8), 29.4 (CH_2_, C-10), 27.3 (CH_3_, C-7'), 22.9 (CH_3_, C-1 6), 18.0 (CH_3_, C-15), 14.5 (CH_3_, C-12). HR-ESIMS *m/z* 374.2335 [M + H]^+^ (calcd for C_22_H_32_NO_4_, 374.2331).

5′-Epiequisetin (**2**)：^1^H NMR (500 MHz, CD_3_OD) *δ*_H_: 5.38~5.42 (2H, m, H-4/5), 5.32 (1H, m, H-13), 5.12 (1H, br s, H-14), 3.94 (2H, overlapped, H_2_-6'), 3.69 (1H, br s, H-5'), 3.35 (1H, overlapped, H-3), 3.04 (3H, s, H_3_-7'), 2.00 (1H, br s, H-10a), 1.89~1.70 (4H, m, H_2_-7, H_2_-9), 1.50 (3H, d, *J* = 6.5 Hz, H-15), 1.48 (3H, overlapped, H_3_-12), 1.10 (2H, m, H-6, H-10b), 0.94 (3H, d, *J* = 7.0 Hz, H_3_-16), 0.86 (1H, m, H-11); ^13^C NMR (125 MHz, CD_3_OD) *δ*_C_: 198.4 (C, C-4'), 192.3 (C, C-1), 178.1 (C, C-2'), 131.9 (CH, C-5/C-13), 131.2 (CH, C-5/C-13), 127.9 (CH, C-4/C-14), 102.4 (C, C-3'), 69.2 (CH, C-5'), 59.4 (CH_2_, C-6'), 49.6 (C, C-2), 46.1 (CH, C-3), 43.7 (CH_2_, C-7), 41.4 (CH, C-11), 40.0 (CH, C-6), 37.0 (CH_2_, C-9), 34.8 (CH, C-8), 29.5 (CH_2_, C-10), 27.3 (CH_3_, C-7'), 22.9 (CH_3_, C-16), 18.0 (CH_3_, C-15), 14.7 (CH_3_, C-12).HR-ESIMS *m/z* 374.2331 [M + H]^+^ (calcd for C_22_H_32_NO_4_, 374.2331).
